# Utilization of pregnant women waiting area and associated factors among mothers at Damboya District, Kembata Tembaro Zone, Southern Ethiopia

**DOI:** 10.11604/pamj.2023.46.89.33071

**Published:** 2023-11-23

**Authors:** Yonas Petros, Sahilu Assegid, Tamrat Shaweno, Melese Markos

**Affiliations:** 1 Public Health expert at Damboya Health Office, Damboya Woreda, Kembata Tembaro Zone, Southern Ethiopia,; 2 Department of Epidemiology, Faculty of Public Health, Institute of Health, Jimma University, Jimma Ethiopia,; 3 African Centers of Diseases Control and Prevention (African CDC), Addis Ababa, Ethiopia,; 4 African Union, Addis Ababa, Ethiopia,; 5 Department of Public Health, College of Health and Medicine Science, Wachamo University, Durame, Ethiopia

**Keywords:** Maternity waiting homes, mothers, utilization, Kembata Tembaro, Ethiopia

## Abstract

**Introduction:**

pregnant women waiting areas are residential facilities where women who live remotely can wait before giving birth at a hospital or health center. About 80% of people in developing countries live in rural areas, where poor access to maternity services accounts for many maternal and perinatal deaths. Although, pregnant women waiting areas are recommended to reduce maternal and infant deaths data on the utilization of pregnant women waiting areas limited in Ethiopia and the study area. Thus, this study assessed the utilization of pregnant mothers waiting area and associated factors among pregnant mothers at Damboya district Kembata Tembaro Zone south, Ethiopia in 2020.

**Methods:**

community-based cross-sectional study was conducted from March 16 to April 15/2020 at Damboya district Kembata Tembaro Zone Southern Ethiopia. Data were collected using a structured interviewer-administered questionnaire. Data were coded, edited, and cleaned then double entered into epi data version 3.1 and exported to SPSS version 20 for analysis. Descriptive, bivariate, and multivariable logistic regression analyses were done. Finally, variables with p-value < 0.05 by multivariate logistic regression analysis were reported as independently associated factors for utilization status of pregnant mothers waiting area.

**Results:**

this study shows pregnant women waiting area utilization was 28.1% at 95%CI (24-32). A distance greater than 30 minutes [AOR: 2.29, 95%CI (1.38-3.62)], wealth fourth quintile [AOR: 3.66, 95%CI (1.73-7.73)], awareness for PWWAs [AOR: 2.30, 95%cCI (1.12,4.74)], Good attitude [AOR: 3.0, 95%CI (1.8-5.0)], Favorable subjective norm [AOR: 2.40, 95%CI (1.50-4.0)] and low perceived barrier [AOR: 2.10, 95%CI (1.30-3.3)] were factors associated with utilization of Pregnant women waiting areas.

**Conclusion:**

utilization of pregnant women waiting areas in the study area was low. A distance greater than 30 minutes, wealthy family, good awareness of pregnant women waiting areas, favorable subjective norm, good attitude, and low perceived barriers were predictors of utilization. To increase their utilization, we need to focus on; improving the economic status of mothers, awareness creation, and work on attitude, subjective norm, and the barriers to utilize pregnant women waiting areas.

## Introduction

Pregnant women waiting areas (PWWAs) are residential facilities where women who live remotely can wait before giving birth at a hospital or health center. The purpose of this home was to provide a setting for women thus can be accommodated during the final weeks of their pregnancy near a hospital with the essential obstetric facility [1]. When access to care is difficult, women with high-risk pregnancies should be admitted to a maternity waiting home (MWH) at 36 weeks of pregnancy [2]. Gradually the concept has been enlarged to include high-risk women including those expecting their first delivery, women with many previous births, very young women, older women, and those identified as having problems such as high blood pressure during pregnancy [3]. Pregnant women waiting homes (PWH) is endorsed by WHO as one of the components of a comprehensive package to reduce maternal morbidity and mortality, which helps to tackle the first and second delays in accessing emergency obstetric care [3,4]. Direct causes of maternal death were considered to be more likely to be promptly diagnosed and treated due to the monitoring of pregnant women in a PWH [5]. In Ethiopia studies from different settings have examined the limited use of PWWA services, and have underlined the need to take local customs [6,7].

The maternal mortality ratio is improperly high internationally and an estimated 42% of maternal death is the intrapartum period [8]. Eight hundred women die from pregnancy or childbirth-related complications around the world every day [9]. In 2015 developing countries report around 99% of the global maternal deaths, with sub-Saharan Africa alone reporting for nearly 66% (200,000), followed by southern Asia 22% [10]. In low-income countries, one woman in 41 dies from maternal causes. One of the major causes of maternal mortality is the distance and consequent delay in the treatment of childbirth complications [10,11]. As the report of EDHS 2016 reveals, Ethiopia has high maternal mortality with an estimated ratio of 412 per 100,000 live births and low skilled birth attendance which is 26% [12] and 48% EMDHS 2019 [13]. PWWA plays an important role in reducing maternal and prenatal mortality by increasing institutional delivery [14]. One of the tested and proven strategies to reduce maternal mortality is the establishment of PWWA practice for pregnant mothers as a component of ANC service, which has been in existence for more than 100 years [15]. PWWA is endorsed by WHO as one of the components of a comprehensive package to reduce maternal morbidity and mortality [3]. Ethiopian MoH recommended the health extension workers to refer the mothers to the waiting houses at the due date/38 weeks of gestation [16].

Although Ethiopia is scaling up PWHs to reduce maternal and perinatal mortality, women´s use of PWHs varies markedly between facilities. Utilization levels of PWHs globally have generally been reported to be low with their conditions often regarded as unsatisfactory [17]. A study conducted in southern Ethiopia, Eastern Gurage reported 15.5% utilization, which implies that institutional delivery is low [18]. According to a study done in the Mettu district, half of the pregnant women intended to use maternity homes [19]. Factors associated with the use of PWHs included longer distances to the nearest health center, higher number of antenatal care visits, higher proportions of complications during ANC, and women's perception of benefits gained from staying in a PWH while waiting for delivery at the health center [20]. Good attitude 55% times, Favorable subjective norm 2.8 times, perceived behavioral control 1.9 times, and giving childbirth in health institutions 1.151 times higher intention to use PWWA [19]. According to studies done in different regions of Ethiopia; occupational status of women, women with a companion to facility visit, wealthier households, those living distant to the health facility, age of women, days stayed less than 15 days, favorable subjective norm and favorable perceived behavior, the suffering complication in previous childbirth were factors associated with using Pregnant women waiting area [6,7,19]. Sustainable development goals by 2030 which is to reduce maternal mortality ratio (MMR) from 420 to 70 per 100,000 live births [21]. Although 100% of health centers have pregnant mothers waiting area according to the 2011 EFY Damboya district health office report institutional delivery service was 69.8% which is low, and also there is no study conducted on PWWA utilization and factors associated. This study aims to assess factors associated with the utilization of PWWAs and to know the magnitude of PWWA utilization. The finding from this study will help to narrow controversial result and enables to work on the factors and have the potential to guide policy-makers to develop a plan for PWWAs in Damboya and beyond also contribute to increasing institutional delivery, reduction in maternal and neonatal mortality.

## Methods

**Study area and period:** the study was conducted in the Damboya district, Kembata Tembaro Zone, in 2019/20 G.C. Damboya district was found in Kembata Tembaro Zone, Southern Ethiopia. It has 20 kebele and it far 350 km from Addis Ababa capital city of Ethiopia and 110 km from Hawassa capital city of SNNP region. It is bounded by East Halaba special District, South Kedida Gamela district, North Shashogo district, and West Angacha district. The total population of the district was 113,469, total pregnancy 3926, and total birth 3926. More than 95% of the population is Kambatissa language speakers and the majority of permanent dwellers were Kembata in ethnicity. The district has a total of 20 health posts, four health centers, and one district hospital. Each health center has a pregnant mother waiting area. The study was conducted from March 16 to April 15/2020.

**Study design:** a community-based cross-sectional study design was used.

**Source population:** all mothers who gave birth in the last 12 months before the study period.

**Study population:** all randomly selected mothers who gave birth 12 months before the study and living in selected kebele during the study period.

**Inclusion and exclusion criteria:** all delivered mothers who lived at least six months in selected kebele were included in this study whereas; all mothers with severe illness and unable to communicate with the interviewer (who were critically ill i.e. couldn't talk or listen) were excluded from the study

**Sample size determination and sampling procedure:** the sample size was calculated using single population proportion formula based on the following assumptions; 5% margin of error, 95% confidence interval, using the proportion of pregnant women waiting area was 31.3% [22]. The sample size was 330. Since, the estimated number of pregnant women in the study area was below 10,000 that is 3926; we used the correction formula to get the actual sample and considering 1.5 design effect and adding 10% none response rate then the final sample which was 501. The Damboya district has 20 kebele, by using simple random sampling seven kebele was selected. The sample size was proportionally allocated to each kebele based on the number of deliveries. To get an individual mothers from each kebele first sampling interval (k) was calculated by dividing the number of mothers in kebele to allocate the sample. The calculated k value was 4. The first mother was identified by lottery method from 1-4 and then based on the random start at each kebele individual mother to be involved were selected by using systematic sampling method starting from the random start at each kebele. A woman in that household had given birth one year before the study period. Whenever more than one eligible woman was found in the same household, one was randomly selected and included in the study.

**Data collection:** the interview questionnaire consists of four parts; Sociodemographic characteristics, cultural, obstetric, and perceived barrier measurement. Data were collected through face-to-face interviews with a pre-tested structured questionnaire, which was adapted by reviewing different kinds of literature [12,19,23]. Seven data collectors, who completed grade ten, can speak Kambatigna and female in sex were recruited as data collectors, and one diploma in midwifery was deployed for the supervisor. They were trained for two days by the principal investigator on the study instrument, consent form, and data collection procedure.

**Variables:** the dependent variable considered in this study was the utilization of pregnant women´s waiting areas. Similarly, independent variables include; *socio-demographic variables:* age, marital status, religion, educational status, occupation, travel time and wealth. *Obstetric factors:* parity, companion for facility use, previous complication, awareness of danger sign, decision-making power, awareness on PWWA, ANC visit, place of delivery, transport. *Cultural factors*: attitude and subjective norm toward PWWA utilization. *Perceived barrier:* stay the mother, stay of companion, upkeep cost, caregiver for family, the mother being away from work, and companion being away from work. *Pregnant women waiting area:* housing facilities positioned within hospitals or health centers to accommodate women in their final weeks of pregnancy up to delivery. *Pregnant women waiting area utilization:* pregnant mothers who were admitted and waited in PWWA during their last pregnancy are considered to be utilized and were coded as 1 and those who never been admitted are not utilized and were coded as 0. *Distance:* this was measured from the report of the mother on walk minutes to a health facility. It was categorized as acceptable ≤ 30 minutes and not acceptable for more than 30 minutes. *Attitude:* the degree to which the person had a favorable or unfavorable evaluation of using PWWA. It was measured by four questions containing five points Likert scale and they were classified into two by using mean as they had good that were greater than or equal to mean score and poor attitude that was less than mean. *Subjective norm:* an individual's perception of using PWWA, which was influenced by the judgment of significant others. It was measured by four questions containing five points Likert scale and they classified into two by using mean as they had favorable who were greater than or equal mean score and unfavorable subjective norm that were less than mean. *Perceived barrier (PB):* the individual´s estimation of the level of challenge to use PWWA. It was measured by six questions containing a five-point Likert scale and they were classified into two by using mean as they have high who were above mean score and low that were less than or equal to mean score of perceived barriers. *Household wealth:* an asset-based wealth index created using the information on asset ownership (radio, television, mobile phone, motorbike, car/truck), number of animals owned (cows, sheep, poultry), electricity supply to the home, drinking water source, type of toilet and type materials used for the construction of floors in the home. Items were coded into relative wealth index using principal component analysis and calibrated into quintiles with each representing 20% of the score from 1(poorest) to 5(richest) quintiles. Mothers were ranked by wealth quintiles by using this index.

**Data processing and analysis:** the data were entered using Epi data version 3.1 statistical software and exported to SPSS version 20 for analysis. Data cleaning was performed to check for frequencies, accuracy, and consistencies, and missed values and variables. The descriptive analysis such as proportions, percentages, means, and measures of dispersion, tables, and graphs was used to describe the data. Bivariate logistic regression was performed to identify candidate variables for multivariate logistic regression. Independent variables at p-value <0.25 in bivariate logistic regression were a candidate for multivariate regression analysis. Multivariate logistic regression analysis was performed by using a backward stepwise method to assess the factors associated. Multicollinearity was checked by using the variance inflation factor (VIF) less than 10 as a cutoff to identify the association between independent variables. The model fitness was checked by the Hosmer-Lemeshow goodness-of-fit statistic test and the result was 0.477. Adjusted odds ratios (AORs) were used to measure the strength of associations and variables with a p-value of less than 0.05 in the multivariable logistic regression analysis were considered as significant associations with the dependent variable.

**Data quality assurance:** to keep data quality the questionnaire (English version) was translated into Kambatigna and translated back to English by two different language experts. Two days of training were given to the data collectors and supervisors on the objective, the relevance of the study, and confidentiality of information, respondent's rights, about pretest, informed consent, and techniques of the interview. A week before actual data collection, the questionnaire was pre-tested on delivered mothers of K/Gamela district on 5 % (25) of the sample by the principal investigator. After pre-testing, amendments were made accordingly. After analyzing data from the pre-test, questions that were not clear were rephrased and corrected. The supervisor and the principal investigator were made frequent checks on the data collection process to ensure the completeness and consistency of the gathered information and errors found during the process were corrected and double-entry verification was done to ensure data quality.

**Ethics approval and consent to participate:** this study was conducted after getting ethical approval from an Institutional Health Research Ethics Review Committee of the Epidemiology Department of Jimma University. Official permission was obtained from the Kembata Tembaro Zone Health department and the Damboye district health office. Informed, written and, signed consent was obtained from the study participants and also parental consent was obtained for those aged less than 18 years old.

## Results

### Socio-demographic characteristics of the respondents

Out of the 501 study participants initially sampled in the study, 495 mothers participated in the study with a response rate of 98.8%. The mean age of the respondents was 28.25 years with ±4.558 SD (standard deviation). A greater part (50%) of the respondents was found in the aged between 20-29 years. Three hundred fifty-five (71.7%) were protestant religious followers. Two hundred eighty-eight (45.5%) of respondents attended primary education. Four hundred eighty-five (98%) were married. Two hundred sixty-five (53.6%) were housewives and ninety-three (20%) of the participants were at the richest quintile (Table 1).

**Table 1:** socio-demographic characteristics of mothers in Damboya district, Kembata Tembaro Zone, South Ethiopia, 2020 (n=495)

Variables	Category	Number	Percent
**Age (in Years)**	**15-19**	13	2.6
	**20-24**	98	19.8
	**25-29**	184	37.2
	**30-34**	163	32.9
	**35 and above**	37	7.5
**Marital Status**	**Married**	485	98
	Other*	10	2
**Education of mother**	**No formal education**	114	23
	**Formal education**	381	77
**Educational status husband**	**No formal education**	43	8.8
	**Formal education**	445	91.2
**Religion**	**Protestant**	355	71.7
	**Muslim**	80	16.2
	**Orthodox**	53	10.7
	**Catholic**	7	1.4
**Occupation**	**Housewife**	419	84.6
	**Merchant**	62	12.5
	Other**	14	2.8
**Time to travel**	**Acceptable (=<30’)**	155	38.8
	**Not acceptable (>30’)**	340	61.2
**Wealth index**	**Poorest**	100	20.2
	**Poor**	92	18.6
	**Medium**	107	21.6
	**Wealthy**	97	19.6
	**Wealthiest**	99	20.0

Note (*=single, widowed and divorced **= Student, Government employee and daily laborers)

### Obstetric factors

Regarding the obstetric factors, 473 (95.6%) had ANC visits and 465 (94%) told complications during pregnancy. Among mothers who were told for complications 370(79.6%) were told abnormal vaginal bleeding, 228(49%) told for swelling of hand and foot, 149(32%) for convulsion. Among all participants 432(87.3%) had two or more children; among mothers who had given birth before only 96(19%) had experienced a pregnancy-related complication; ninety-five percent had given birth at the health institution (Table 2).

**Table 2:** obstetric factors in Damboya district, Kembata Tembaro Zone, Southern Ethiopia, 2020 (n=495)

Variables	Category	Number	Percent
**Antenatal Care**	**No**	22	4.4
	**Yes**	473	95.6
**Parity**	**One child**	63	12.7
	**Two or more**	432	87.3
**Companion during Delivery**	**No**	35	7.1
	**Yes**	460)	92.9
**Awareness of danger sign during pregnancy**	**No**	30	6.1
	**Yes**	465	93.9
**Experienced complication during a recent delivery**	**No**	369	74.5
	**Yes**	96	19.4
**Place of recent delivery**	**Health facility**	477	96
	**Home**	18	4
**Transportation problem to reach the health facility**	**No**	470	94.6
	**Yes**	25	5.1
**Decision-making maternal and child health at the household level**	**Not involved**	163	32.9
	**Involved**	332	67.1

### Cultural and perceived barrier and Service received at pregnant mothers waiting area

Regarding attitude towards PWWA use, 297(60%) respondents reported that they had a good attitude towards the utilization of PWWA. Two hundred eighty-three (57.2%) respondents agreed that they had favorable subjective norms. On the other hand, among the total respondents who were assessed for perceived barriers two hundred fifteen (43.4%) of respondents had a low perceived barrier. Among mothers who received the service 98 (70.6%) were satisfied with the service provided at PWWA. More than three-fourths of mothers received food, kitchen utensils, and drinking water at the pregnant women waiting area in the study area (Table 3).

**Table 3:** attitude toward PWWA utilization, subjective norm, and perceived barriers of mothers in Damboya district, Kembata Tembaro Zone, southern Ethiopia, 2020 (n=495)

Variable	Category	Frequency	Percent
**Attitude to PWWA utilization**	Poor	198	40.0
	Good	297	60.0
**Subjective norm**	Unfavorable	211	42.6
	Favorable	284	57.4
**Food**	No	20	14.4
	Yes	119	85.6
**Kitchen utensils**	No	15	10.8
	Yes	124	89.2
**The place for the stay of companion**	No	90	64.7
	Yes	49	35.3
**Electric light**	No	60	43.2
	Yes	79	56.8
**Drinking water**	No	34	24.6
	Yes	105	75.4
**Perceived Barriers**	High	280	56.6
	Low	215	43.4

### Service utilization status

The overall pregnant women waiting area utilization in the study area was 139 (28.1%) at 95%CI (24-32) (Figure 1). Among all the mothers who used PWWAs 22(15.8%) stayed for more than fifteen days. Among mothers who used PWWA, 79.9% used it because of good health outcomes, 71.9% referred from health posts by HEWs, 11.5% because they used it previously, and 10.1% were recommended, other family members.

### Factors associated with PWWA utilization

In the bivariable logistic regression: age of the respondents, mother's occupation, time to reach HF, household wealth, place of birth, awareness of danger sign, transportation to HF, having companion, decision-making power, awareness of PWWA, attitude, subjective norm, and the perceived barrier were candidate variables for multivariate logistic regression (Table 4). In multivariate logistic regression living at a distance greater than 30 minutes on the walk is two times more likely to utilize PWWAs AOR=2.29, 95%CI 1.38, 3.6. Wealthy families were 3.6 times more likely to utilize PWWAs when compared with the poorest wealth index (first quintile) of the family AOR =3.66, (95%CI) = (1.73, 7.73). Women who have a good awareness of PWWAs were greater than two times more likely to utilize PWWAs (AOR =2.3, (95%CI) = (1.12, 4.74)). Those who had a good attitude were three times more likely to utilize PWWAs AOR =3.11, 95%CI: 1.842, 5.26. The mother who had favorable subjective norm greater than two times more likely to utilize PWWAs (AOR =2.4, (95%CI) (1.5, 4)) and mothers with low perceived barriers 2.1 times more likely to utilize pregnant women waiting area (AOR = 2.1, (95%CI) (1.30, 3.30)) were factors associated significantly at p-value less than 0.05 (Table 4).

**Table 4:** factors associated with PWWA among mothers in Damboya district, Kembata Tembaro Zone, Southern Ethiopia, 2020 (n=495)

Variables	Category	Utilization		COR (95% C.I)	AOR (95%CI)
		Yes	No		
**Age (year)**	**15-19**	6(46.2%)	7(53.8%)	1	1
	**20-24**	29(29.6%)	69(70.4%)	.490(.15, 1.59)	0.45(0.12,1.60)
	**25-29**	44(23.9 %)	140(76.1%)	.37(.12, 1.15)	0.40(0.11,1.39)
	**30-34**	51(31.3%)	112(68.7%)	.53(.17, 1.66)	0.49(0.14,1.69)
	**35 and above**	9(24.3%)	28(75.7%)	.38(.10, 1.41)	0.41(0.98,1.75)
**Mothers Occupation**	**Housewife**	121(28.9)	298(71. %)	1	1
	**Merchant**	13(21%)	49(79%)	0.65(.34, 1.25)	0.76(0.37,1.55)
	**Other**	5(35.7%)	9(64.3%)	1.54(.49,4.79)	0.99(0.21,4.61)
**Time to reach HF**	**30 minutes**	31(20%)	124(80%)	1	1
	**> 30 minutes**	108(31.8%)	232(68.2%)	1.86(1.18, 2.94)	2.29(1.38,3.62)*
**Wealth index**	**Poorest**	17(17%)	83(83%)	1	1
	**Poor**	21(22.8%)	71(72.2%)	1.444(.71, 2.95)	1.4(.66, 3)
	**Medium**	32(29.9%)	75(70.1%)	2.08(1.070,4.05)	2.25(1.1,4.7)
	**Wealthy**	38(39.2%)	59(60.8%)	3.145(1.62,6.09)	3.66(1.73,7.73)
	**Wealthiest**	31(31.3%)	68(68.7%)	2.23(1.14,4.36)	2.25(1.06, 4.76)
**Awareness to danger sign**	**No**	5(16.7%)	25(83.3%)	1	1
	**Yes**	134(28.8%)	331(71.2%)	2.02(0.76,5.39)	1.42(0.42,4.74)
**Experience complication**	**No**	102(27.6%)	267(72.4%)	1	1
	**Yes**	32(33.3%)	64(66.7%)	1.31(0.81, 2.12)	1.10(0.63,1.95)
**Transportation problem**	**No**	134(28.8%)	332(71.2%)	1	1
	**Yes**	5(17.2%)	24(81.8%)	0.52(0.19, 1.38)	0.27(0.12,1.04)
**Companion to visit HF**	**No**	6(17.1%)	29(82.9%)	1	1
	**Yes**	133(28.9%)	327(71.1%)	1.97(.79, 4.84)	1.18(0.35,4.00)
**Awareness on PWWA**	**Poor**	11(11.2%)	87(88.8%)	1	1
	**Good**	128(32.2%)	269(67.8%)	3.76(1.94,7.29)	2.30(1.12,4.74)*
**Attitude**	**Unfavorable**	25(12.6%)	173(87.4%)	1	1
	**Favorable**	114(38.4%)	183(61.6%)	4.31(2.67,6.97)	3.00(1.8,5.0)**
**Subjective norm**	**Unfavorable**	32(15.2%)	179(84.8%)	1	1
	**Favorable**	107(37.7%)	177(62.8%)	3.38(2.16,5.28)	2.40(1.5, 4.0)**
**Perceived barrier**	**High**	56(20%)	224(80%)	1	1
	**Low**	83(38.6%)	132(61.4%)	2.52(1.68,3.76)	2.10(1.3, 3.3)**
**Decision making**	**Not Involved**	37(22.7%)	126(77.3%)	1	1
	**Involved**	102(30.7%)	230(69.3%)	1.51(0.98,2.33)	1.03(0.59,1.76)

Note (*p<0.05, **p<0.001, COR = Crude Odd Ratio, AOR=Adjusted odd ratio, CI= Confidence interval, HF = Health Facility, PWWA = Pregnant Women Waiting Area)

## Discussion

Different studies were conducted on PWWAs services availability and intention to use. This study mainly focused on the utilization and factors associated with it. Wealth index, time to travel, attitude, subjective norm, perceived barrier, and awareness of PWWAs were identified as predictors of utilization. In this study, 28.1% of respondents had used PWWAs in 12 months before the study period. This result was comparable with the study in Tanzania and Zambia which were 31.3% and 31.5% respectively [22,24,25]. Also, this study was comparable with a study conducted in Zambia which reported 29.4% of mothers to stay at PWWA [20]; but this study was lower when compared to a study conducted at Gomma Woreda of Jimma Zone in 2016 which is 38.7% of respondents had past MWH experience [23]. This difference could be due to the study was facility-based and the objective of the study and the study population was different from this study. This study finding was higher when compared with the study conducted from 2014-2018 facilitators for maternity waiting home (MWH) utilization which reported among mothers who attended birth 15.5% used MWH [18]. This difference could be due to the variation in the study period and sampling method which consecutive.

In this study, we found that the majority of women in our study were aware of the existence of PWWAs but a very small proportion reported having used the service. A cross-sectional study conducted in Jimma Zone, Ethiopia, reported just 71% of women interviewed being aware of PWWA services compared with 80.2% in this study [6]. Most women accessed PWWAs through referrals from health extension workers during antenatal care and generally stayed at PWWAs for less than 15 days before delivering their babies. The relatively short duration of stay suggests that many users are women who may be presented with false labor and are accommodated temporarily at the PWWAs; this may be because they are not being referred to PWWAs 2 weeks before delivery as recommended [16]. This may partially explain why community norms around facility deliveries were not significantly associated with PWWA use. Therefore, referral practices around, and promotion of, PWWA use employed by HEWs and health workers in the area require investigation to ensure that the women who would benefit the most from this service are being reached. According to this study, those who are in the wealthy quintile are around 4 times more likely to utilize PWWAs compared with the poorest ones; Despite PWWA services being free, there may be financial and social costs associated with lodging there. Women from wealthier households are probably more likely to be able to afford to pay for transport, purchase food, and accommodate accompanying relatives. This finding is also similar to a study that has a finding from Nepal that reported women from wealthier families are more likely to utilize [26]. Also, another study done on determinants of utilization of maternal health care services among pregnant women in Ghana Kwaku south district reported household income was a significantly associated predictor of utilization [27]. Some studies have reported an inverse relationship between MWH use and household wealth [28,29] and after adjusting for confounders [22].

Time to travel greater than 30 minutes to a health facility is another predictor of utilization of pregnant women waiting area with around 2 times more likely to utilize PWWAs compared to living within 30 minutes, this is comparable with a study conducted in 2016 at Jimma Zone Ethiopia [6] and study report of Tigray region in 2015 living within ≥30 min is 47% times less likely to utilize skilled delivery service [30]. It is therefore not surprising to find that women who report living within 30 min of a health facility are less inclined to use PWWA services. Large distances between homes and health facilities are often part of PWWA admission criteria [16,31]. Studies in Africa have reported distance from health facilities affecting women's decisions to use PWWA services [22,32]. According to this study respondents with good attitudes were 3 times more likely to use PWWAs; this is comparable with the study report conducted in the 2014 Kalomo district, Zambia [20]. A study was done in Zambia also revealed that attitudes influenced the use of maternal health care services [28].

Subjective norm is one of a predictor of utilization of the pregnant women’s waiting area. In this study, women with favorable Subjective norms were more than two times more likely to use PWWAs. This is comparable with a study done in the Mettu district, the subjective norm as a predictor of intention to use PWWA. This is lower when compared with the study report conducted in the 2014 Kalomo district, Zambia this difference could be due to the socio-economic difference of the country [20]. Forty-three percent of respondents have a low perceived barrier which significantly associated with PWWAs utilization with two times more likely than that of the high perceived barrier, this finding is comparable with the EMONC survey report in 2015 which 45% of respondents had not reported a barrier to utilize MWA [4]. Also, this finding is similar to a study done in 2014 in SNNPR eastern Gurage which reported envisioning relatively few barriers was associated with utilization of PWWAs [7]. According to a study done in the Mettu district perceived barrier was also a predictor of intention to use PWWA [19].

**Limitations of the study:** the cross-sectional nature of the analysis does not support causal inference limiting this to an exploratory exercise to identify factors that may influence PWWA use. Also, the primary outcome relied on women´s self-reported PWWAs' use which may be subject to recall bias. On the other hand, this risk is likely low because staying at a PWWA before delivery is expected to be a remarkable experience. Women's self-reported travel times estimates may not exactly reflect the physical accessibility of PWWAs; calculation of distances is recommended for future studies to assess the distance threshold for PWWAs utilization.

## Conclusion

The magnitude of the utilization of pregnant women waiting area among mothers in the study area was low. Time to travel, wealth, awareness of PWWAs, attitude, subjective norm, and perceived barriers were factors associated with utilization. Regular and Close supervision and monitoring activities should be done to ensure PWWA services are provided as per the standards and registering and reporting mechanism and Integration with health extension workers, community, and HDA leaders should be strengthened to reduce the influences of important other barriers of utilization. Advocating of kebele leaders and HDA leaders should be done to mobilize resources. Promoting partner involvement to support utilization and health education to the community on PWWAs.

### What is known about this topic

- *Factors associated with utilization of pregnant women waiting area were assessed in Damboya district, some of the factors identified were distance greater than 30 minutes, wealthy family, good awareness of pregnant women waiting areas, favorable subjective norm, good attitude, and low perceived barriers*.

### What this study adds

- *This study assesses the level of utilization of pregnant mothers waiting area;*
- *The factors of among pregnant mothers for utilization of pregnant women waiting area at Damboya district;*
- *Unlike age of the respondents, mother occupation, place of birth, awareness of danger sign, transportation to HF, having companion, decision making power were did not associated with pregnant women waiting area*.
